# Intensive Blood Pressure Control and Cardiovascular Outcomes Across Cardiovascular-Kidney-Metabolic Syndrome Stages: A Post Hoc Analysis of the China Rural Hypertension Control Project

**DOI:** 10.1001/jamanetworkopen.2025.57180

**Published:** 2026-02-13

**Authors:** Xiaofan Guo, Shiyu Zhou, Jianjun Mu, Chunxia Zhao, Guozhe Sun, Ying Zhou, Yao Yu, Xiangyu Tan, Yangzhi Yin, Ziyi Xie, Wei Miao, Wenhang Li, Caiyu Zhang, Chenhua He, Jie Chen, Xiaoxuan Tian, Tianhao Li, Yifei Chen, Xiaobing Zhou, Mengling Lu, Qiyu Li, Ning Ye, Guangxiao Li, Yingxian Sun

**Affiliations:** 1 Department of Cardiology, the First Hospital of China Medical University, Shenyang, Liaoning, China; 2 Department of Clinical Epidemiology and Evidence-Based Medicine, The First Hospital of China Medical University, Shenyang, China; 3 Department of Cardiovascular Medicine, First Affiliated Hospital of Xi’an Jiaotong University, Xi’an, China; 4 Division of Cardiology, Department of Internal Medicine, Tongji Hospital, Tongji Medical College of Huazhong University of Science and Technology, Wuhan, China; 5 Department of Medical Record Management Center, the First Hospital of China Medical University, Shenyang, China

## Abstract

**Importance:**

Cardiovascular-kidney-metabolic (CKM) syndrome represents a syndromic continuum encompassing overlapping cardiovascular, kidney, and metabolic dysfunction. Hypertension is a central driver in the pathogenesis of CKM syndrome, promoting both kidney and metabolic deterioration, but little is known about the benefits of intensive blood pressure (BP) control across CKM syndrome stages.

**Objective:**

To evaluate CKM syndrome stage–specific outcomes, safety, and net clinical benefits associated with a comprehensive intensive BP intervention.

**Design, Setting, and Participants:**

This is a post hoc, secondary analysis of a cluster randomized clinical trial, the China Rural Hypertension Control Project (CRHCP), which was conducted between May 2018 and March 2023. Participants were adults aged 40 years or older with hypertension and CKM syndrome stages 2 to 4, which were defined using standard criteria. Stage 2 indicates that the patient has a metabolic risk factor; stage 3, subclinical cardiovascular disease or predicted 10-year risk of 20% or greater; and stage 4, clinical cardiovascular disease. In this analysis, participants had a median (IQR) follow-up of 3.02 (2.97-3.06) years. Data analysis was conducted from November 2024 to June 2025.

**Intervention:**

The comprehensive BP control strategy targeted a systolic BP less than 130 mm Hg and a diastolic BP less than 80 mm Hg. The intervention was delivered by trained nonphysician practitioners.

**Main Outcomes and Measures:**

The primary clinical outcome was a composite of major adverse cardiovascular events (stroke, myocardial infarction, heart failure, or cardiovascular death). Safety outcomes included hypotension, syncope, injurious falls, and kidney adverse events. A quantitative benefit-harm analysis was conducted to estimate net benefit associated with the intervention.

**Results:**

Among 33 736 participants (mean [SD] age, 63.0 [9.2] years; 20 677 [61.3%] women), 18 662 (55.3%) had stage 2 CKM syndrome (of whom 9526 [51.0%] received the intervention), 7984 (23.7%) had stage 3 (of whom 4032 [50.5%] received the intervention), and 7090 (21.0%) had stage 4 (of whom 3713 [52.4%] received the intervention). Treatment outcomes were generally consistent across CKM syndrome stages. Intensive BP control was associated with reduced cardiovascular events across all stages: in stage 2, the hazard ratio (HR) was 0.61 (95% CI, 0.50-0.73); in stage 3, the HR was 0.71 (95% CI, 0.58-0.84); and in stage 4, the HR was 0.67 (95% CI, 0.58-0.76). The risk of all-cause mortality was also lower in stage 2 (HR, 0.73; 95% CI, 0.57-0.90) and stage 3 (HR, 0.82; 95% CI, 0.68-0.96) but not in stage 4 (HR, 1.02; 95% CI, 0.84-1.20). Risk of hypotension increased across all stages (relative risk range, 1.79-2.34), while other adverse events, including kidney events, were similar between groups, despite some numerical variation across stages. Net benefits were favorable: stage 2, 1.58 (95% CI, 1.53-1.62); stage 3, 2.53 (95% CI, 2.42-2.64); and stage 4, 2.15 (95% CI, 2.04-2.26).

**Conclusions and Relevance:**

In this post hoc analysis of a cluster randomized clinical trial, a comprehensive intervention targeting BP less than 130/80 mm Hg demonstrated a favorable benefit-to-harm profile across CKM syndrome stages, with no clear heterogeneity across CKM syndrome stages. These findings provide the first trial-based evidence to guide CKM syndrome management and support scalable strategies for this high-risk, multimorbid population.

**Trial Registration:**

ClinicalTrials.gov Identifier: NCT03527719

## Key Points

**Question:**

Is intensive blood pressure management associated with consistent net clinical benefits across cardiovascular-kidney-metabolic (CKM) syndrome stages?

**Findings:**

In a secondary analysis of a large cluster randomized trial involving more than 33 000 adults, a comprehensive intensive blood pressure intervention was associated with significantly reduced risk of cardiovascular events across CKM syndrome stages 2 to 4. Net benefit analyses demonstrated favorable benefit-to-harm profiles at all stages.

**Meaning:**

This study offers the first trial-based evidence to guide stage-specific CKM syndrome care and supports scalable strategies for high-risk, multimorbid populations, particularly in resource-limited settings, such as rural China.

## Teaser

This secondary analysis of a randomized clinical trial evaluates whether there are any differences in the outcomes, safety, and net clinical benefits associated with an intensive blood pressure control intervention by cardiovascular-kidney-metabolic (CKM) syndrome stages.

## Introduction

Cardiovascular-kidney-metabolic (CKM) syndrome, newly defined by the American Heart Association (AHA) in 2023,^1^ captures the syndromic convergence of cardiovascular disease (CVD), chronic kidney disease (CKD), and type 2 diabetes (T2D). These conditions often coexist and interact, leading to worse cardiovascular outcomes and substantial public health and economic burdens.^2,3,4,5^ The CKM syndrome framework stages individuals from 0 to 4 based on risk factors, organ dysfunction, and established disease,^1^ shifting chronic disease management from an organ-specific approach toward integrated, system-level strategies.

The prevalence of CKM syndrome is striking. In a nationally representative US cohort of 10 762 adults, approximately 90% met criteria for stage 1 or higher, and 15% were classified as stage 3 or higher.^6^ In China, nationally representative data from 2012 to 2015 showed that more than 80% of adults fell into stage 1 or higher, with nearly 1 in 4 reaching the advanced stages of CKM syndrome (stages 3-4), underscoring a substantial and growing public health burden.^7^ Despite its high prevalence and clinical complexity, current research provides limited evidence to guide risk-stratified management across CKM syndrome stages, particularly in clinical settings.

Hypertension is a central driver in the pathogenesis of CKM syndrome, promoting both kidney and metabolic deterioration. Evidence from high-risk populations shows that intensive blood pressure (BP) control can reduce cardiovascular morbidity and mortality by 24%,^8^ while each 5–mm Hg reduction in systolic BP in patients with CKD yields an additional 10% relative risk (RR) reduction.^9^ Hypertension also contributes to CKD onset and progression, substantially increasing the risk of end-stage kidney disease (ESKD).^10^ Moreover, it is closely linked to metabolic dysfunction: individuals with poorly controlled BP are significantly more likely to have metabolic syndrome and T2D than those with effective BP control, as demonstrated by the Global Cardiometabolic Risk Profile Survey.^11^

Although multiple randomized trials have established the cardiovascular benefits of intensive BP lowering in patients with hypertension,^12,13,14,15,16,17^ it remains unknown whether these benefits—and the associated risks—differ across stages of CKM syndrome. The China Rural Hypertension Control Project (CRHCP) was a large-scale, cluster-randomized trial that evaluated a comprehensive BP management strategy targeting systolic BP less than 130 mm Hg and diastolic BP less than 80 mm Hg, delivered by trained nonphysician practitioners in rural China.^14,15^ In this post hoc analysis, we assessed the cardiovascular outcomes, safety, and stage-specific net clinical benefit associated with intensive BP control across CKM syndrome stages. To our knowledge, this is the first trial-based investigation to stratify treatment benefit and harm across CKM syndrome stages, providing critical evidence to inform stage-specific clinical decision-making and policy design in CKM syndrome population management.

## Methods

### Design and Participants

This study is a post hoc subgroup analysis based on CKM syndrome staging within the CRHCP. The CRHCP was an open-label, blinded end point, cluster randomized clinical trial conducted from May 2018 to March 2023 across 326 villages in 3 provinces of rural China. A total of 33 995 adults aged 40 years or older with hypertension were enrolled, with 17 407 participants from 163 villages assigned to the intervention group and 16 588 from another 163 villages receiving usual care. There were no strict exclusion criteria, enhancing the generalizability of the findings. The trial evaluated the effectiveness and safety of a comprehensive, intensive BP management strategy targeting BP less than 130/80 mm Hg, delivered by trained nonphysician community health care practitioners. Detailed descriptions of the trial design, implementation, and primary outcomes have been published previously.^18,19^ The trial was approved by the Ethics Committee of the First Hospital of China Medical University and all participating institutions. Written informed consent was obtained from all participants at enrollment. The protocol and statistical analysis plan of CRHCP are available in Supplement 1. This analysis was conducted post hoc and was not prespecified in the protocol. This report follows the Consolidated Standards of Reporting Trials (CONSORT) reporting guideline for randomized clinical trials.

Of the 33 995 participants enrolled in the CRHCP trial, 259 were excluded due to missing data required for CKM syndrome staging. Because the proportion of missing data was small, no data imputation was performed. Information on participants lost to follow-up has been reported in the original CRHCP trial. A total of 33 736 participants were included in the present analysis (eFigure 1 in Supplement 2). CKM syndrome was staged from 0 to 4 based on the 2023 AHA framework and prior literature from a Korean population.^1,20^ Because all participants in the CRHCP trial were diagnosed with hypertension at enrollment, no participants were in stages 0 or 1. This post hoc analysis thus focused on the higher-risk CKM syndrome stages (stages 2-4). Stage 2 was defined as the presence of a metabolic risk factors (eg, hypertriglyceridemia, defined as triglyceride level of 133 mg/dL or greater [to convert to micromoles per liter, multiply by 0.0113], metabolic syndrome, diabetes) or moderate to high–risk CKD based on Kidney Disease: Improving Global Outcomes (KDIGO) criteria, in the absence of clinical CVD and with a 10-year predicted total CVD risk of less than 20%. Stage 3 included participants with subclinical CVD, such as very high-risk CKD per KDIGO or a predicted 10-year CVD risk of 20% or greater, but without clinical CVD. Stage 4 was defined as clinical CVD, including self-reported coronary heart disease, myocardial infarction, or stroke, and represents the full expression of CKM syndrome. Estimated glomerular filtration rate (eGFR) was calculated using the 2021 Chronic Kidney Disease Epidemiology Collaboration creatinine equation. The risk of CVD was derived from the 10-year atherosclerotic cardiovascular disease risk estimated using the pooled cohort equations proposed by the American College of Cardiology and the AHA. The full staging definitions are provided in eTable 1 in Supplement 2.

### Intervention and Measurements

The CRHCP was a cluster randomized clinical trial. The intervention group received a comprehensive intensive BP management strategy targeting a goal of less than 130/80 mm Hg. The strategy was designed to address barriers at multiple levels—health care systems, practitioners, patients, and communities—and was implemented by trained nonphysician community health care practitioners. These individuals underwent standardized training and operated under the supervision of primary care physicians. They were responsible for initiating and titrating antihypertensive therapy according to a streamlined, protocol-driven stepped-care algorithm. In addition to pharmacologic management, the intervention included structured health coaching focused on home BP monitoring, lifestyle modification, medication adherence, and control of cardiovascular risk factors. To improve treatment adherence, participants in the intervention group were also offered access to free or low-cost antihypertensive medications (eTable 2 in Supplement 2).

Baseline demographic data were collected via structured questionnaires, and sex was self-reported. Laboratory evaluations were conducted at both baseline and 36 months. Follow-up assessments occurred every 6 months and were performed by trained study staff. At each visit, BP was measured 3 times with participants seated and at rest for at least 5 minutes, in accordance with standardized protocols. Measurements were taken using an automated oscillometric device (Omron HBP-1100U [Omron Corp]), with appropriately sized cuffs selected based on arm circumference. BP data were transmitted in real time via mobile devices to the centralized study data center to ensure data fidelity.

### Study Outcome

The primary clinical outcome was a composite of major cardiovascular events, comprising myocardial infarction, stroke, heart failure, and cardiovascular death. Each component of this composite, as well as all-cause mortality, was also analyzed.

The primary safety outcome was defined as the occurrence of any adverse event of interest, including injurious falls, hypotension, syncope, ESKD or dialysis, a 50% or greater decline in eGFR among participants with CKD at baseline, or a 30% or greater decline in eGFR to less than 60 mL/min/1.73 m^2^ among those without CKD at baseline.

### Statistical Analysis

We performed intention-to-treat analyses to compare study outcomes between the intervention and control groups, based on village-level randomization and irrespective of individual adherence. The association of the intervention with outcomes was stratified by CKM syndrome stages, with potential heterogeneity of effect examined through testing the interactions of CKM syndrome × treatment on outcomes. To estimate hazard ratios (HRs) and 95% CIs for cardiovascular events, marginal Cox models were used, with villages as random effects. To account for multiple testing across outcomes, we applied false discovery rate (FDR) correction to enhance the robustness of the results. In these models, sex, age, province, county, township, treatment group, CKM syndrome stage, and the interaction term for CKM syndrome stage and treatment were included as fixed effects, and a robust sandwich variance estimator was applied to account for clustering at the village level.

To evaluate the trade-off between benefit and harm, we conducted a quantitative benefit-harm analysis. The absolute risk reduction (ARR) for cardiovascular outcomes was calculated as the difference in cumulative event incidence between the control and intervention groups. Conversely, the absolute risk increase (ARI) for adverse events was estimated similarly. First, preliminary weights for benefit and harm outcomes were derived from a structured patient preference survey conducted among individuals with hypertension by Johns Hopkins University and Kaiser Permanente Colorado.^21,22^ These weights reflected patient-perceived severity, burden, and acceptability of various clinical outcomes. Second, a multidisciplinary expert panel consisting of 11 members, including cardiologists, neurologists, a nephrologist, an epidemiologist, a health policy expert, and patient representatives, reviewed and refined these initial weights to ensure clinical relevance and alignment with stakeholder perspectives. Third, a targeted literature review was conducted to validate the consistency of the selected weights with published estimates of outcome severity and patient values.^23,24,25^ The final weights used in the net benefit analysis are detailed in eTable 3 in Supplement 2. Mean weights were calculated for the cardiovascular benefit end points and total adverse events of interest, resulting in a relative weight ratio of 1:3.6. Net clinical benefit was calculated as: Net Benefit = ARR − (ARI × weight).

We conducted several sensitivity analyses to test the robustness of our findings. First, we used multivariable-adjusted Cox models adjusting for baseline covariates including age, sex, smoking status, antihypertensive medication use, history of CVD, diabetes, baseline systolic BP, and low-density lipoprotein (LDL) cholesterol level. Second, a propensity score matching (PSM) strategy matched 16 465 intensive–usual care pairs (1:1 nearest-neighbor, without replacement) using age, sex, antihypertensive drug use, systolic and diastolic BP, diabetes status, body mass index, history of CVD, and LDL cholesterol level as matching factors; covariate balance was assessed by standardized mean differences, with values less than 0.1 indicating good balance. Third, we assessed alternative weighting schemes, including increasing the relative weight of harm and applying a 1:5 benefit-to-harm ratio. In addition, we plotted the estimated net benefit across a range of weight ratios (from 1:1 to 1:10) to visualize how different assumptions about benefit-harm preferences affected the results. Fourth, we applied a Fine-Gray subdistribution hazard model to account for the competing risk of death.

All statistical tests were 2-sided, and *P* < .05 was considered statistically significant. Analyses were conducted using R version 4.2.0 (R Project for Statistical Computing).

## Results

### Baseline Characteristics

A total of 33 736 participants (mean [SD] age, 63.0 [9.2] years; 20 677 [61.3% women]) were included in this analysis, comprising 18 662 (55.2%) with CKM syndrome stage 2 (of whom 9526 [51.0%] received the intervention), 7984 (23.7%) with stage 3 (of whom 4032 [50.5%] received the intervention), and 7090 (21.0%) with stage 4 (of whom 3713 [52.4%] received the intervention) (Table 1). Compared with those with stage 2, participants in stages 3 and 4 were generally older, more often male, and more likely to be taking antihypertensive medications. They also had higher systolic BP, LDL cholesterol, fasting glucose, and uric acid levels as well as lower diastolic BP levels and eGFR. Within each CKM syndrome stage, baseline characteristics were generally balanced between the intensive and usual care groups. Table 1 summarizes the baseline characteristics of participants by CKM syndrome stage and intervention group.

**Table 1.** Baseline Characteristics by CKM Syndrome Stage of Participants

Baseline characteristics	Participants, mean (SD)					
	CKM syndrome stage 2		CKM syndrome stage 3		CKM syndrome stage 4	
	Intervention (n = 9526)	Usual care (n = 9136)	Intervention (n = 4032)	Usual care (n = 3952)	Intervention (n = 3713)	Usual care (n = 3377)
Age, y	58.4 (7.4)	58.9 (7.4)	71.7 (6.7)	71.9 (6.9)	64.5 (8.0)	64.9 (8.1)
Sex, No. (%)						
Female	6910 (72.5)	6727 (73.6)	1623 (40.3)	1587 (40.2)	1992 (53.6)	1838 (54.4)
Male	2616 (27.5)	2409 (26.4)	2409 (59.7)	2365 (59.8)	1721 (46.4)	1539 (45.6)
Education, No. (%)						
Primary school or less	5823 (61.1)	5907(64.7)	2991 (74.2)	2907 (73.6)	2590 (69.8)	2414 (71.5)
Junior high school	3025 (31.8)	2643 (28.9)	850 (21.1)	876 (22.2)	926 (24.9)	765 (22.7)
High school	583 (6.1)	520 (5.7)	175 (4.3)	150 (3.8)	186 (5.0)	184 (5.4)
College or higher	95 (1.0)	66 (0.7)	16 (0.4)	19 (0.5)	11 (0.3)	14 (0.4)
Smoking, No. (%)						
Never smoked	7564 (79.4)	7252 (79.4)	2190 (54.3)	2033 (51.4)	2393 (64.4)	2162 (64.0)
Former smokers	515 (5.4)	518 (5.7)	421 (10.4)	398 (10.1)	504 (13.6)	492 (14.6)
Current smokers	1447 (15.2)	1366 (15.0)	1421 (35.2)	1521 (38.5)	816 (22.0)	723 (21.4)
Weekly alcohol drinking, No. (%)	1478 (15.5)	1357 (14.9)	878 (21.8)	941 (23.8)	431 (11.6)	389 (11.5)
Physical activity ≥5 times/wk, No. (%)^a^	5384 (56.5)	5279 (57.8)	1724 (42.8)	1761 (44.6)	1405 (37.8)	1256 (37.2)
Use of antihypertensive medications, No. (%)	5445 (57.2)	4624 (50.6)	2441 (60.5)	2111 (53.4)	2675 (72.0)	2240 (66.3)
Body mass index^b^	26.5 (3.9)	26.3 (3.8)	25.1 (3.8)	24.9 (3.8)	25.8 (3.8)	25.7 (3.7)
Waist circumference, cm	89.6 (9.7)	88.9 (9.7)	88.4 (10.1)	87.5 (10.2)	89.5 (9.8)	88.9 (9.7)
Systolic blood pressure, mm Hg	154.0 (16.0)	152.7 (15.4)	163.5 (19.2)	162.2 (18.6)	157.3 (19.2)	155.2 (18.5)
Diastolic blood pressure, mm Hg	88.9 (10.3)	88.3 (10.0)	86.2 (11.3)	85.4 (11.3)	87.9 (10.9)	86.8 (10.8)
Total cholesterol, mg/dL	194.9 (37.9)	194.4 (37.7)	196.7 (39.7)	197.1 (40.4)	193.2 (42.3)	191 (41.5)
LDL cholesterol, mg/dL	103.6 (30.7)	103.5 (30.4)	107.3 (32.7)	107.4 (33.2)	105.2 (33.9)	103.4 (32.7)
HDL cholesterol, mg/dL	56.8 (13.4)	56.5 (13.1)	55.3 (14.0)	55.3 (14.0)	54.1 (12.8)	53.8 (12.8)
Triglycerides, median (IQR), mg/dL	141.7 (98.3-209.0)	139.9 (96.5-207.3)	128.4 (89.5-189.5)	125.8 (88.6-195.7)	139.9 (97.4-207.3)	134.6 (94.8-204.6)
Fasting plasma glucose, mg/dL	109 (34.1)	108.8 (34.0)	114.6 (40.2)	113.7 (37.9)	113.3 (38.9)	112.5 (37.1)
Uric acid, mg/dL	5.0 (1.5)	5.0 (1.4)	5.3 (1.4)	5.3 (1.4)	5.2 (1.4)	5.2 (1.4)
eGFR, mL/min/1.73 m^2^^c^	96.5 (11.8)	95.8 (11.7)	84.6 (13.1)	84.1 (13.0)	90.0 (14.0)	89.6 (13.7)

Abbreviations: CKM, cardiovascular-kidney-metabolic; eGFR, estimated glomerular filtration rate; HDL, high-density lipoprotein; LDL, low-density lipoprotein.

SI conversion factors: To convert total, HDL, and LDL cholesterol to millimoles per liter, multiply by 0.0259; triglycerides to millimoles per liter, multiply by 0.0113; glucose to millimoles per liter, multiply by 0.0555; and uric acid to millimoles per liter, multiply by 0.0595.

^a^ Moderate or heavy physical activity for at least 30 minutes at a time.

^b^ Body mass index is calculated as weight in kilograms divided by height in meters squared.

^c^ eGFR was calculated based on the 2021 Chronic Kidney Disease Epidemiology Collaboration creatinine equations.

### BP During Follow-Up

As shown in eFigure 2 in Supplement 2, participants in the intervention groups experienced a more pronounced and sustained reduction in both systolic and diastolic BP across all CKM syndrome stages compared with those receiving usual care. By 36 months, net reductions in systolic BP ranged from 21.4 to 26.2 mm Hg and in diastolic BP from 9.8 to 9.9 mm Hg across CKM syndrome stages 2 to 4. Regarding antihypertensive medication use (eTable 4 in Supplement 2), participants in the intervention groups were more likely to receive treatment, particularly with angiotensin converting enzyme inhibitors/angiotensin receptor blockers and calcium channel blockers, across all CKM syndrome stages.

### Clinical Outcomes

At a median follow-up of 3.02 years, intensive BP management was associated with significantly reduced risk of major cardiovascular events across all CKM syndrome stages. All interaction *P* values were greater than .05, indicating no evidence of heterogeneity across stages. The HRs were 0.61 (95% CI, 0.50-0.73) for stage 2, 0.71 (95% CI, 0.58-0.84) for stage 3, and 0.67 (95% CI, 0.58-0.76) for stage 4. All-cause mortality was also significantly lower in the intervention group in stage 2 (HR, 0.73; 95% CI, 0.57-0.90) and stage 3 (HR, 0.82; 95% CI, 0.68-0.96) but not in stage 4 (HR, 1.02; 95% CI, 0.84-1.20) (Table 2). For individual cardiovascular outcomes, numerical differences across stages were observed, but none of the interaction tests were significant. Stroke risk declined consistently across stages (HRs of 0.61 [95% CI, 0.48-0.73], 0.67 [95% CI, 0.53-0.81], and 0.69 [95% CI, 0.58-0.79] for stages 2, 3, and 4, respectively). Cardiovascular mortality showed the largest relative reduction in stage 2 (HR, 0.46; 95% CI, 0.25-0.66), with similar directions but 95% CIs that contained the null for stages 3 and 4. Kaplan-Meier curves confirmed these patterns across CKM syndrome stages (Figure; eFigures 3-6 in Supplement 2). The intraclass correlation coefficients were small for all outcomes within each CKM syndrome stage (eTable 5 in Supplement 2).

**Table 2.** Risk of Cardiovascular Outcomes and All-Cause Mortality Associated With Intensive Blood Pressure Treatment, According to CKM Syndrome Stages^a^

Outcomes	CKM syndrome stage 2		CKM syndrome stage 3		CKM syndrome stage 4		*P* for interaction
	Hazard ratio (95%CI)	*P* value	Hazard ratio (95%CI)	*P* value	Hazard ratio (95%CI)	*P* value	
Major cardiovascular outcomes	0.61 (0.50-0.73)	<.001	0.71 (0.58-0.84)	<.001	0.67 (0.58-0.76)	<.001	.53
Myocardial infarction	0.65 (0.31-0.98)	.15	1.00 (0.60-1.40)	.99	0.68 (0.38-0.99)	.15	.93
Stroke	0.61 (0.48-0.73)	<.001	0.67 (0.53-0.81)	<.001	0.69 (0.58-0.79)	<.001	.53
Heart failure	0.65 (0.12-1.18)	.45	0.80 (0.30-1.30)	.49	0.47 (0.21-0.74)	.03	.53
Death from cardiovascular causes	0.46 (0.25-0.66)	.002	0.76 (0.51-1.00)	.13	0.81 (0.58-1.04)	.14	.12
Death from all causes	0.73 (0.57-0.90)	.02	0.82 (0.68-0.96)	.04	1.02 (0.84-1.20)	.85	.10

Abbreviation: CKM, cardiovascular-kidney-metabolic.

^a^ The model adjusted for fixed effects including sex, age, province, county, township, treatment group, CKM syndrome stage, and the CKM syndrome stage × treatment interaction term, with village included as a random effect. *P* values were adjusted using the Benjamini-Hochberg false discovery rate method to account for multiple comparisons.

**Figure.**
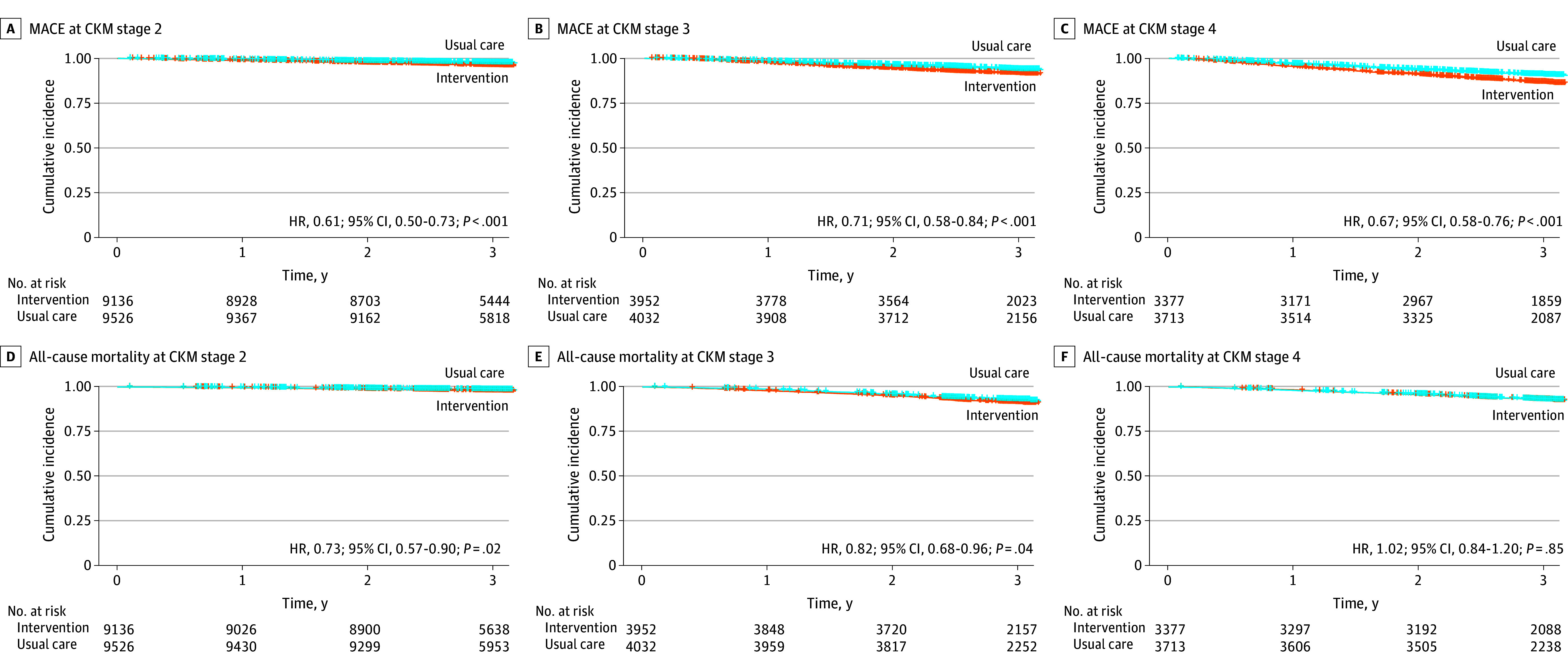
Kaplan-Meier Curves for Major Adverse Cardiovascular Events (MACE) and All-Cause Mortality in Patients With Different Cardiovascular-Kidney-Metabolic (CKM) Syndrome Stages HR indicates hazard ratio.

Adverse events were assessed across CKM syndrome stages (Table 3). Patterns of safety outcomes were broadly consistent across stages, with no statistically significant interactions. The incidence of hypotension was higher in all intervention groups compared to usual care, with RRs of 1.79 (95% CI, 1.31-2.26; *P* < .001) in stage 2, 2.34 (95% CI, 1.40-3.29; *P* < .001) in stage 3, and 2.23 (95% CI, 1.16-3.30; *P* = .001) in stage 4. Kidney adverse events were numerically higher in stage 3 (RR, 1.53; 95% CI, 1.04-2.01), but this association did not reach statistical significance after adjustment for multiple comparisons (*P* = .06). The incidence of other adverse events, including injurious falls and syncope, was similar between groups across all stages.

**Table 3.** Safety and Kidney Outcomes Associated With Intensive Blood Pressure Treatment According to CKM Syndrome Stages^a^

Outcomes	CKM syndrome stage 2		CKM syndrome stage 3		CKM syndrome stage 4		*P* for interaction
	Risk ratio (95%CI)	*P* value	Risk ratio (95%CI)	*P* value	Risk ratio (95%CI)	*P* value	
Total adverse events of interest	1.28 (1.06-1.51)	.03	1.75 (1.31-2.19)	<.001	1.33 (1.01-1.66)	.03	.92
Injurious fall	0.98 (0.54-1.42)	.92	1.69 (0.64-2.74)	.30	0.91 (0.38-1.44)	.92	.66
Hypotension	1.79 (1.31-2.26)	<.001	2.34 (1.40-3.29)	<.001	2.23 (1.16-3.30)	.001	.66
Syncope	0.94 (0.36-1.53)	.86	1.10 (0.30-1.91)	.79	1.46 (0.40-2.53)	.31	.79
Kidney adverse events^b^	1.03 (0.71-1.36)	.98	1.53 (1.04-2.01)	.06	1.05 (0.66-1.43)	.98	.66

Abbreviation: CKM, cardiovascular-kidney-metabolic.

^a^ The model adjusted for fixed effects including sex, age, province, county, township, treatment group, CKM syndrome stage, and the CKM syndrome stage × treatment interaction term, with village included as a random effect. *P* values were adjusted using the Benjamini-Hochberg false discovery rate method to account for multiple comparisons.

^b^ Kidney adverse events included end-stage kidney disease or dialysis, a 50% or greater reduction in estimated glomerular filtration rate in patients with chronic kidney disease at baseline, or a 30% or greater reduction in estimated glomerular filtration rate to less than 60 mL/min/1.73 m^2^ in patients without chronic kidney disease at baseline.

### Net Benefit Associated With Intensive Treatment

Intensive BP control led to ARRs in major cardiovascular events of 1.85% (95% CI, 1.81%-1.89%) in stage 2, 2.92% (95% CI, 2.82%-3.03%) in stage 3, and 2.45% (95% CI, 2.34%-2.55%) in stage 4 over the 3.02-year follow-up period, largely driven by a decline in stroke incidence (Table 4). At the same time, the ARIs in adverse events were 0.97% (95% CI, 0.94%-1.01%), 1.41% (95% CI, 1.33%-1.50%), and 1.07% (95% CI, 1.00%-1.14%), respectively. When combining both benefits and harms using weighted outcomes, the net clinical benefit remained positive across all CKM syndrome stages. The estimated net benefit values were 1.58 (95% CI, 1.53-1.62) in stage 2, 2.53 (95% CI, 2.42-2.64) in stage 3, and 2.15 (95% CI, 2.04-2.26) in stage 4.

**Table 4.** Net Benefit Associated With Intensive Treatment According to CKM Syndrome Stages^a^

Outcome	ARD		
	CKM syndrome stage2	CKM syndrome stage3	CKM syndrome stage4
**Benefit, ARR (95% CI), %**			
Major cardiovascular outcomes^b^	1.85 (1.81-1.89)	2.92 (2.82-3.03)	2.45 (2.34-2.55)
Myocardial infarction	0.14 (0.13-0.15)	0.26 (0.24-0.28)	0.20 (0.18-0.22)
Stroke	1.58 (1.55-1.61)	2.29 (2.21-2.37)	2.02 (1.94-2.09)
Heart failure	0.13 (0.12-0.14)	0.29 (0.28-0.31)	0.21 (0.19-0.22)
Death from cardiovascular causes	0.31 (0.29-0.33)	0.87 (0.81-0.93)	0.55 (0.50-0.60)
Death from all causes	0.43 (0.39-0.47)	1.28 (1.08-1.48)	0.77 (0.61-0.93)
**Harm, ARI (95% CI), %**			
Total adverse events of interest^c^	0.97 (0.94-1.01)	1.41 (1.33-1.50)	1.07 (1.00-1.14)
Injurious fall	0.03 (0.03-0.05)	0.05 (0.04-0.06)	0.04 (0.03-0.05)
Hypotension	0.79 (0.77-0.82)	0.95 (0.91-0.99)	0.86 (0.83-0.89)
Syncope	0.02 (0.01-0.03)	0.05 (0.03-0.08)	0.29 (0.06-0.50)
Kidney adverse events^d^	0.17 (0.15-0.19)	0.32 (0.27-0.38)	0.16 (0.12-0.20)
**Benefit vs harm, ARD (95% CI)**			
Major cardiovascular outcomes vs total adverse events of interest	1.58 (1.53-1.62)	2.53 (2.42-2.64)	2.15 (2.04-2.26)

Abbreviations: ARD, absolute risk difference; ARI, absolute risk increase; ARR, absolute risk reduction; CKM, cardiovascular-kidney-metabolic.

^a^ Weights were determined through a 3-step adjudication process, with 1 cardiovascular benefit equating to 3.6 total adverse events of interest. Net benefit was calculated as the absolute risk reduction minus the absolute risk increase × weights.

^b^ Major cardiovascular events included myocardial infarction, stroke, heart failure, and cardiovascular death.

^c^ Total adverse events of interest included injurious fall, hypotension, syncope, and kidney adverse events.

^d^ Kidney adverse events included end-stage kidney disease or dialysis, a 50% or greater reduction in estimated glomerular filtration rate in patients with chronic kidney disease at baseline, or a 30% or greater reduction in estimated glomerular filtration rate to <60 mL/min/1.73 m^2^ in patients without chronic kidney disease at baseline.

### Subgroup and Sensitivity Analyses

Forest plots demonstrated that results for both major cardiovascular events and all-cause mortality were consistent across all subgroups within each CKM syndrome stage (eFigures 7 and 8 in Supplement 2). We further evaluated net clinical benefit stratified by age, sex, and educational level (eFigure 9 in Supplement 2). In all CKM syndrome stages, participants aged 60 years and older and male participants were associated with greater net benefits compared with younger or female participants. Sensitivity analyses using multivariable adjustment, PSM, alternative 1:5 weighting, and a competing risk model yielded results consistent with the primary analysis (eTable 6 in Supplement 2). Across sensitivity analyses that varied the relative weighting between benefits and harms from 1:1 to 1:10, intensive BP control consistently yielded a positive net benefit across all CKM syndrome stages (eFigure 10 in Supplement 2).

## Discussion

In this post hoc analysis of the CRHCP, we evaluated the stage-specific outcomes, safety, and net clinical benefit associated with intensive BP control among patients with CKM syndrome. Intensive control was associated with significantly reduced risk of major cardiovascular events across all CKM syndrome stages and showed consistent directional reductions in all-cause mortality, which reached statistical significance in stages 2 and 3, with no clear heterogeneity across CKM syndrome stages. Net benefit analyses revealed favorable benefit-to-harm results in all stages. To our knowledge, this is the first study to quantify the stage-specific clinical value of intensive BP management in the CKM syndrome population.

Prior studies have established CKM syndrome staging as a robust predictor of cardiovascular and all-cause mortality,^26,27,28^ particularly in advanced stages (3-4), where event rates are substantially higher. These outcomes are driven by a multifactorial pathophysiology involving systemic inflammation,^29^ metabolic dysregulation,^30,31^ and adverse social and behavioral determinants.^26,32^ While intensive BP lowering has shown cardiovascular benefit in patients with metabolic syndrome,^33,34,35^ its risk-benefit profile across the spectrum of CKM syndrome had not been previously characterized to our knowledge. In this study, we addressed this gap by evaluating both efficacy and safety across CKM syndrome stages and by quantifying the overall net clinical benefit using an integrated benefit-to-harm framework, which showed consistently favorable results in all stages (1.58 in stage 2, 2.53 in stage 3, and 2.15 in stage 4). These findings provide a more nuanced basis for stage-informed, risk-adapted BP management in the expanding CKM syndrome population.

Interestingly, we observed notable differences across CKM syndrome stages in baseline characteristics. Participants in stage 3 were older and had higher systolic blood pressure, LDL cholesterol, and fasting glucose compared with other stages, consistent with prior population-based studies showing greater clustering of metabolic and kidney abnormalities in CKM syndrome stage 3.^32,36,37,38^ These differences may reflect the distinct staging criteria, with stage 3 defined by predicted cardiometabolic risk and stage 4 by established CVD, and likely represent the accumulation of risk factors before overt clinical disease. Because stage 3 carried a higher burden and variability of risk factors, multivariable adjustment in sensitivity analyses modestly attenuated the effect estimates, although the overall direction of treatment benefit remained unchanged.

Although relative treatment effects were generally consistent across CKM syndrome stages, some variation in outcome patterns was observed. Prior studies have similarly shown comparable or even lower mortality in stage 3 relative to stage 4.^27,38^ In our analysis, intensive BP control was associated with reduced risk of cardiovascular events across all stages, while the mortality benefit reached statistical significance only in stages 2 and 3, likely reflecting differences in disease reversibility and competing non-CVD risks in more advanced stages. Stage-specific differences in end points, such as reduced CV death in stage 2 and lower heart failure incidence in stage 4, likely arise from different dominant mechanisms across the CKM syndrome continuum, with atherosclerotic event prevention being more prominent in earlier stages and hemodynamic or neurohormonal effects predominating in advanced CVD.^39,40^

Intensive BP control is often associated with adverse effects, such as hypotension, syncope, electrolyte disturbances, and kidney impairment,^33,41,42,43,44,45^ with these risks particularly evident in individuals with diabetes, CKD, or metabolic syndrome. In our analysis, hypotension risk increased consistently across all CKM syndrome stages, whereas kidney adverse events tended to be higher in stage 3, likely reflecting greater hemodynamic sensitivity and reduced kidney autoregulatory reserve at this intermediate stage.^46^ The RR for injurious falls was greater than 1 in stage 3 (although it did not reach statistical significance), possibly due to older age and increased susceptibility to orthostatic BP changes.^47^ Despite these differences, the overall balance of benefits and harms remained favorable, supporting the broad applicability of intensive BP control across the CKM syndrome spectrum.

Current CKM syndrome management guidelines lack stage-specific evidence to guide BP-lowering strategies.^48,49^ Our findings provide trial-based support for implementing intensive BP control across CKM syndrome stages through a risk-stratified net benefit approach. Notably, the intervention in this study was delivered by trained nonphysician practitioners within a community setting,^14^ underscoring its scalability and feasibility. This integrated, nonphysician-led model offers a pragmatic and sustainable framework for CKM syndrome management, especially in resource-limited settings. By linking emerging CKM syndrome staging frameworks with an actionable intervention, this study helps inform future clinical guidelines and health system strategies for multimorbid, high-risk populations.

### Limitations

This study has several limitations. First, as a post hoc analysis of the CRHCP trial, residual confounding may exist despite adjustment strategies. Second, the operational definition of CKM syndrome staging was based on available clinical data and lacked imaging or biomarker-based validation,^50^ which may affect stage classification accuracy. However, collecting such detailed data is often infeasible in large community-based trials. Similar definitions have been adopted in previous CKM syndrome–related population studies, ensuring comparability of our findings.^27,28,30,32,36,37,38,51^ Future studies incorporating imaging, biomarker, and mechanistic data are warranted to achieve more precise CKM syndrome staging. In addition, all study sites were rural primary care facilities with broadly similar diagnostic capacity. Therefore, the potential for systematic bias in CKM syndrome stage classification due to regional differences in diagnostic resources is considered low. Third, all participants had baseline hypertension, corresponding to at least stage 2 in the CKM syndrome framework, limiting our ability to evaluate earlier stages. Fourth, conducted in rural, resource-limited settings, this study provides a scalable model for intensive BP management that may be applicable to similar low-resource contexts globally. Nevertheless, caution is warranted when extrapolating these findings to high-resource or urban settings, where health system structures and patient characteristics may differ.

## Conclusions

In conclusion, this is the first study of which we are aware to evaluate an intensive BP management strategy across clinically defined stages of CKM syndrome. A comprehensive, nonphysician-led intervention targeting BP levels less than 130/80 mm Hg was associated with significantly reduced risk of cardiovascular events at all CKM syndrome stages. Although certain adverse events were elevated, the overall net clinical benefit remained favorable. These findings provide the first trial-based, stage-specific evidence to inform CKM syndrome management and support scalable, clinical strategies for this high-risk, multimorbid population.

## Notes

Supplement 1.
Trial Protocol

Supplement 2. **eTable 1.** Framework for CKM Syndrome Staging in This Study **eTable 2.** Intervention Strategies for Intervention Group and Usual Care Group **eTable 3.** Weights of Benefit-Harm Trade-Off Outcomes in the Study **eTable 4.** Types of Antihypertensive Medications Used in Participants Between Different Stages of CKM Syndrome **eTable 5.** Intraclass Correlation Coefficients in Different CKM Stages **eTable 6.** Sensitivity Analyses of Net Benefit **eFigure 1.** Flowchart of the Study **eFigure 2.** Blood Pressure Over the 36 Months of Follow-Ups in Patients With Different CKM Syndrome Stages **eFigure 3.** Kaplan-Meier Curves for Myocardial Infarction in Patients With Different CKM Syndrome Stages **eFigure 4.** Kaplan-Meier Curves for Stroke in Patients With Different CKM Syndrome Stages **eFigure 5.** Kaplan-Meier Curves for Heart Failure in Patients With Different CKM Syndrome Stages **eFigure 6.** Kaplan-Meier Curves for Cardiovascular Death in Patients With Different CKM Syndrome Stages **eFigure 7.** Forest Plot of Major Cardiovascular Outcomes According to Subgroups in Participants With Different CKM Syndrome Stages **eFigure 8.** Forest Plot of All-Cause Death According to Subgroups in Participants With Different CKM Syndrome Stages **eFigure 9.** Forest Plot of Net Benefits According to Subgroups in Participants With Different CKM Syndrome Stages **eFigure 10.** Net Benefit Analysis Comparing Intensive and Standard Blood Pressure Control With Different Weights for Harm Outcomes

Supplement 3.
Data Sharing Statement
