# Supplementary material for: Intensive Blood Pressure Control and Cardiovascular Outcomes Across Cardiovascular-Kidney-Metabolic Syndrome Stages: A Post Hoc Analysis of the China Rural Hypertension Control Project
Source: JAMA Netw Open. 2026 Feb 13;9(2):e2557180. doi: 10.1001/jamanetworkopen.2025.57180 (PMC12905657; doi:10.1001/jamanetworkopen.2025.57180)
Supplement: Supplement 2. — eTable 1. Framework for CKM Syndrome Staging in This Study eTable 2. Intervention Strategies for Intervention Group and Usual Care Group eTable 3. Weights of Benefit-Harm Trade-Off Outcomes in the Study eTable 4. Types of Antihypertensive Medications Used in Participants Between Different Stages of CKM Syndrome eTable 5. Intraclass Correlation Coefficients in Different CKM Stages eTable 6. Sensitivity Analyses of Net Benefit eFigure 1. Flowchart of the Study eFigure 2. Blood Pressure Over the 36 Months of Follow-Ups in Patients With Different CKM Syndrome Stages eFigure 3. Kaplan-Meier Curves for Myocardial Infarction in Patients With Different CKM Syndrome Stages eFigure 4. Kaplan-Meier Curves for Stroke in Patients With Different CKM Syndrome Stages eFigure 5. Kaplan-Meier Curves for Heart Failure in Patients With Different CKM Syndrome Stages eFigure 6. Kaplan-Meier Curves for Cardiovascular Death in Patients With Different CKM Syndrome Stages eFigure 7. Forest Plot of Major Cardiovascular Outcomes According to Subgroups in Participants With Different CKM Syndrome Stages eFigure 8. Forest Plot of All-Cause Death According to Subgroups in Participants With Different CKM Syndrome Stages eFigure 9. Forest Plot of Net Benefits According to Subgroups in Participants With Different CKM Syndrome Stages eFigure 10. Net Benefit Analysis Comparing Intensive and Standard Blood Pressure Control With Different Weights for Harm Outcomes [file jamanetwopen-e2557180-s002.pdf]

Supplemental Online Content

Guo X, Zhou S, Mu J, et al. Net benefit of intensive blood pressure control across cardiovascular-kidney-metabolic syndrome stages: a post hoc analysis of the china rural hypertension control project. *JAMA Netw Open.* 2026;9(2):e2557180.
doi:10.1001/jamanetworkopen.2025.57180

eTable 1. Framework for CKM Syndrome Staging in This Study

eTable 2. Intervention Strategies for Intervention Group and Usual Care Group

eTable 3. Weights of Benefit-Harm Trade-Off Outcomes in the Study

eTable 4. Types of Antihypertensive Medications Used in Participants Between Different Stages of CKM Syndrome

eTable 5. Intraclass Correlation Coefficients in Different CKM Stages

eTable 6. Sensitivity Analyses of Net Benefit

eFigure 1. Flowchart of the Study

eFigure 2. Blood Pressure Over the 36 Months of Follow-Ups in Patients With Different CKM Syndrome Stages

eFigure 3. Kaplan-Meier Curves for Myocardial Infarction in Patients With Different CKM Syndrome Stages

eFigure 4. Kaplan-Meier Curves for Stroke in Patients With Different CKM Syndrome Stages

eFigure 5. Kaplan-Meier Curves for Heart Failure in Patients With Different CKM Syndrome Stages

eFigure 6. Kaplan-Meier Curves for Cardiovascular Death in Patients With Different CKM Syndrome Stages **eFigure 7.** Forest Plot of Major

© 2026 Guo X et al. *JAMA Network Open*.

Cardiovascular Outcomes According to Subgroups in Participants With Different CKM Syndrome Stages

eFigure 8. Forest Plot of All-Cause Death According to Subgroups in Participants With Different CKM Syndrome Stages

eFigure 9. Forest Plot of Net Benefits According to Subgroups in Participants With Different CKM Syndrome Stages

eFigure 10. Net Benefit Analysis Comparing Intensive and Standard Blood Pressure Control With Different Weights for Harm Outcomes

This supplemental material has been provided by the authors to give readers additional information about their work.

© 2026 Guo X et al. *JAMA Network Open*.

eTable 1. Framework for CKM Syndrome Staging in This Study

CKM stages	Criterion	Threshold for CKM conditions	
Stage 2: Metabolic risk factors and CKD	Individuals with metabolic risk factors (hypertriglyceridemia, hypertension, MetS, diabetes), or Moderate- to high-risk CKD according to Kidney Disease: Improving Global Outcomes criteria.	Any of the five criteria is met	Hypertriglyceridemia
			Hypertension
			Diabetes
			MetS
			Moderate- to high-risk CKD
		All criteria are met	Predicted 10-year total CVD risk < 20%
			No clinical CVD
Stage 3: Subclinical CVD in CKM	Individuals with very-high-risk CKD according to Kidney Disease: Improving Global Outcomes criteria or a high predicted 10-year CVD risk.	Any of the two criteria is met	Very-high-risk CKD
			Predicted 10-year total CVD risk ≥ 20%
		All criteria are met	No clinical CVD
Stage 4: Clinical CVD in CKM	Individuals with self-reported CVD (coronary heart disease, heart failure, or stroke)	All criteria are met	Clinical CVD

Abbreviations: CKM= cardiovascular-renal-metabolic, CKD= Chronic Kidney Disease, MetS= Metabolic Syndrome, CVD= Cardiovascular Disease.

© 2026 Guo X et al. *JAMA Network Open*.

eTable 2. Intervention Strategies for Intervention Group and Usual Care Group

Group	Strategies
Intervention group	**Health Systems**<br>• Developed structured infrastructure for chronic disease management<br>• Incorporated hypertension control rates into performance-based resource allocation<br>• Provided free or low-cost antihypertensive medications<br><br>**Non-Physician Community Health-Care Providers**<br>• Trained to measure blood pressure using standardized protocols<br>• Trained to follow a simplified stepwise algorithm for BP management<br>• Equipped to deliver lifestyle counseling and adherence support<br><br>**Patients**<br>• Trained to monitor home blood pressure accurately<br>• Encouraged to adopt healthy lifestyle behaviors and maintain medication adherence<br>• Engaged in peer support groups to enhance motivation and continuity
Usual care group	Non-physician community health-care providers only receive training in standardised blood pressure measurements

© 2026 Guo X et al. *JAMA Network Open*.

eTable 3. Weights of Benefit-Harm Trade-Off Outcomes in the Study

Outcome	Event type	Weight
**Benefit**	Major cardiovascular event	0.77
	Myocardial infarction	0.66
	Stroke	0.76
	Heart failure	0.65
	Death from cardiovascular causes	1
**Harm**	Total adverse events of interest	0.21
	Injurious fall	0.01
	Hypotension	0.11
	Syncope	0.02
	End-stage renal disease or dialysis	0.47
	≥50% reduction in eGFR in patients with chronic kidney disease at baseline or ≥30% reduction in eGFR to <60 mL/min per 1.73 m² in patients without chronic kidney disease at baseline	0.45

Abbreviations: eGFR= estimated Glomerular Filtration Rate.

© 2026 Guo X et al. *JAMA Network Open*.

eTable 4. Types of Antihypertensive Medications Used in Participants Between Different Stages of CKM Syndrome

Antihypertensive medications	Stage2		Stage3		Stage4	
	Intervention	Usual care	Intervention	Usual care	Intervention	Usual care
No. of patients who reported taking antihypertensive medications during the past two weeks, n (%)	8815 (92.5)	6831 (74.8)	3540 (87.8)	2781 (70.4)	3257 (87.7)	2552 (75.6)
ACE inhibitors/angiotensin II receptor blockers, n (%)	7152 (75.1)	2730 (29.9)	2937 (72.8)	1017 (25.7)	2674 (72.0)	940 (27.8)
Beta blockers, n (%)	106 (1.1)	123 (1.3)	46 (11.4)	44 (11.1)	50 (1.3)	51 (1.5)
Calcium channel blockers, n (%)	7730 (81.1)	3424 (37.5)	3246 (80.5)	1485 (37.6)	2935 (79.0)	1463 (43.3)
Diuretics, n (%)	5849 (61.4)	765 (8.4)	2382 (59.1)	272 (6.9)	2122 (57.2)	232 (6.9)
Others, n (%)	2 (0.1)	703 (7.7)	1 (0.1)	318 (8.0)	1 (0.1)	233 (6.9)

Abbreviation: CKM= cardiovascular-renal-metabolic, ACE= angiotensin converting enzyme.

ACE inhibitors/angiotensin II receptor blockers: Benazepril, Candesartan, Captopril, Enalapril, Fosinopril, Imidapril, Irbesartan, Lisinopril, Losartan, Olmesartan, Perindopril, Ramipril, Telmisartan, and Valsartan. Beta blockers: Arotinolol, Atenolol, Bisoprolol, Carvedilol, Labetalol, Metoprolol, and Propranolol. Calcium channel blockers: Amlodipine, Benidipine, Cinildipine, Diltiazem, Felodipine, Lacidipine, Lercanidipine, Nifedipine, Nimodipine, Nitrendipine, and Verapamil. Diuretics: Furosemide, Hydrochlorothiazide, Indapamide, Spirolactone, and Triamterene. Others: Beijing compound antihypertensive tablets (reserpine-dihydralazine-hydrochlorothiazide-triamterene), Compound reserpine tablets (reserpine-hydrochlorothiazide-dihydralazine-promethazine), Compound antihypertensive tablets (reserpine-dihydralazine-hydrochlorothiazide), Zhenju compound antihypertensive tablets (clonidine-hydrochlorothiazide), Compound triazine and rutinum tablets (reserpine-dihydralazine-hydrochlorothiazide), Compound bendazol hydrochlorothiazide capsules (reserpine-hydrochlorothiazide), Compound tetrazine reserpine tablets (reserpine-dihydralazine-hydrochlorothiazide), Compound apocynum (dihydralazine-hydrochlorothiazide), Reserpine, Dihydralazine, Clonidine, and Urapidil. All types of antihypertensive drug including compound formulation will be analyzed separately. Data are numbers (percentages). The sums of proportions are over 100% due to patients who took multiple antihypertensive medications.

© 2026 Guo X et al. *JAMA Network Open*.

eTable 5. Intraclass Correlation Coefficients in Different CKM Stages

Outcomes	Stage2	Stage3	Stage4
**Major cardiovascular outcomes**	0.007	0.011	0.021
Myocardial infarction	0.004	0.018	0.021
Stroke	0.005	0.007	0.003
Heart failure	0.002	0.004	0.000
Death from cardiovascular causes	0.001	0.007	0.021
**Death from all causes**	0.002	0.015	0.002

Abbreviation: CKM= cardiovascular-renal-metabolic

eTable 6. Sensitivity Analyses of Net Benefit

	Net benefit (%)		
**Sensitivity analyses**	**Stage2**	**Stage3**	**Stage4**
Multi-adjusted model	1.16 (1.12-1.21)	2.16 (2.03-2.27)	3.79 (3.55-4.04)
Propensity score matching	1.60 (1.55-1.65)	2.61 (2.50-2.72)	2.17 (2.05-2.29)
Weighted 1:5	1.65 (1.61-1.70)	2.64 (2.53-2.75)	2.23 (2.12-2.34)
Competing risk	1.56 (1.51-1.61)	2.46 (2.34-2.55)	2.09 (2.00-2.23)

The full model was additionally adjusted for: smoking status, baseline antihypertensive medication use, history of cardiovascular disease, baseline systolic blood pressure, fasting blood glucose and low-density lipoprotein cholesterol. Propensity score matching strategy successfully matched a total of 16465 pairs of patients in the two groups.

© 2026 Guo X et al. *JAMA Network Open*.

eFigure 1. Flowchart of the Study

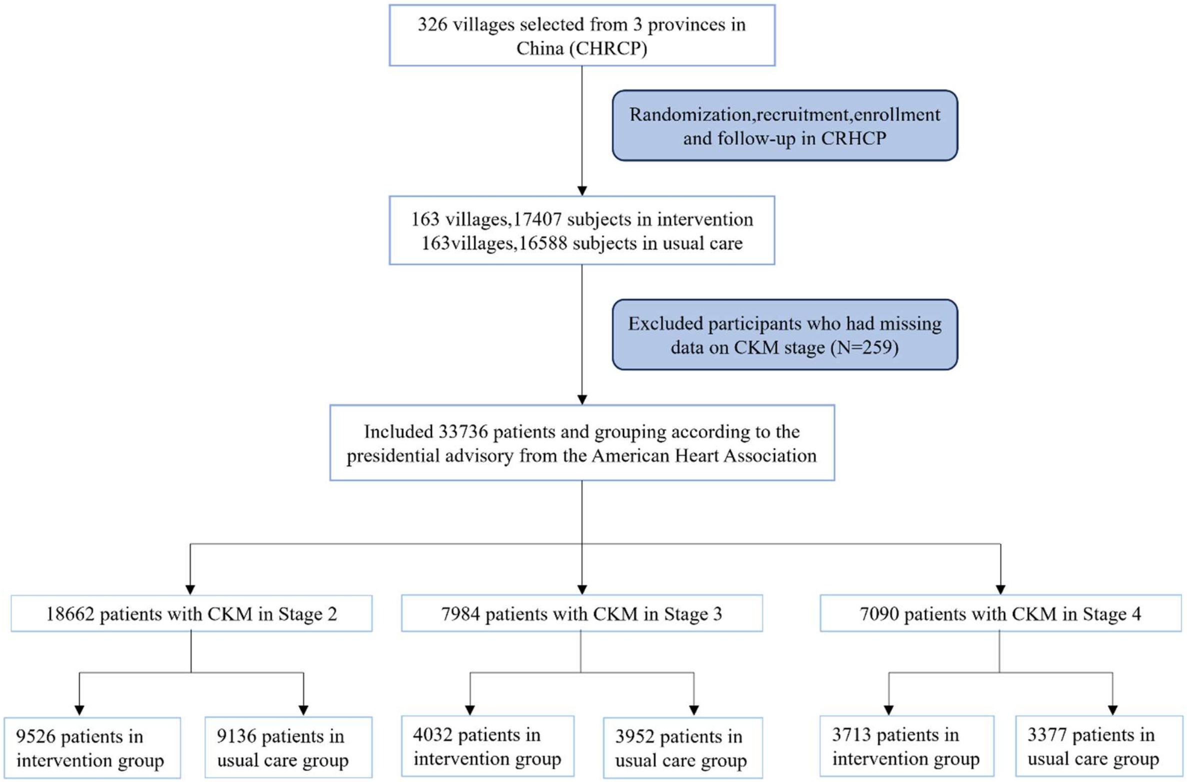

Abbreviation: CKM= cardiovascular-renal-metabolic. CHRCP= China Rural Hypertension Control Program.

© 2026 Guo X et al. *JAMA Network Open*.

eFigure 2. Blood Pressure Over the 36 Months of Follow-Ups in Patients With Different CKM Syndrome Stages

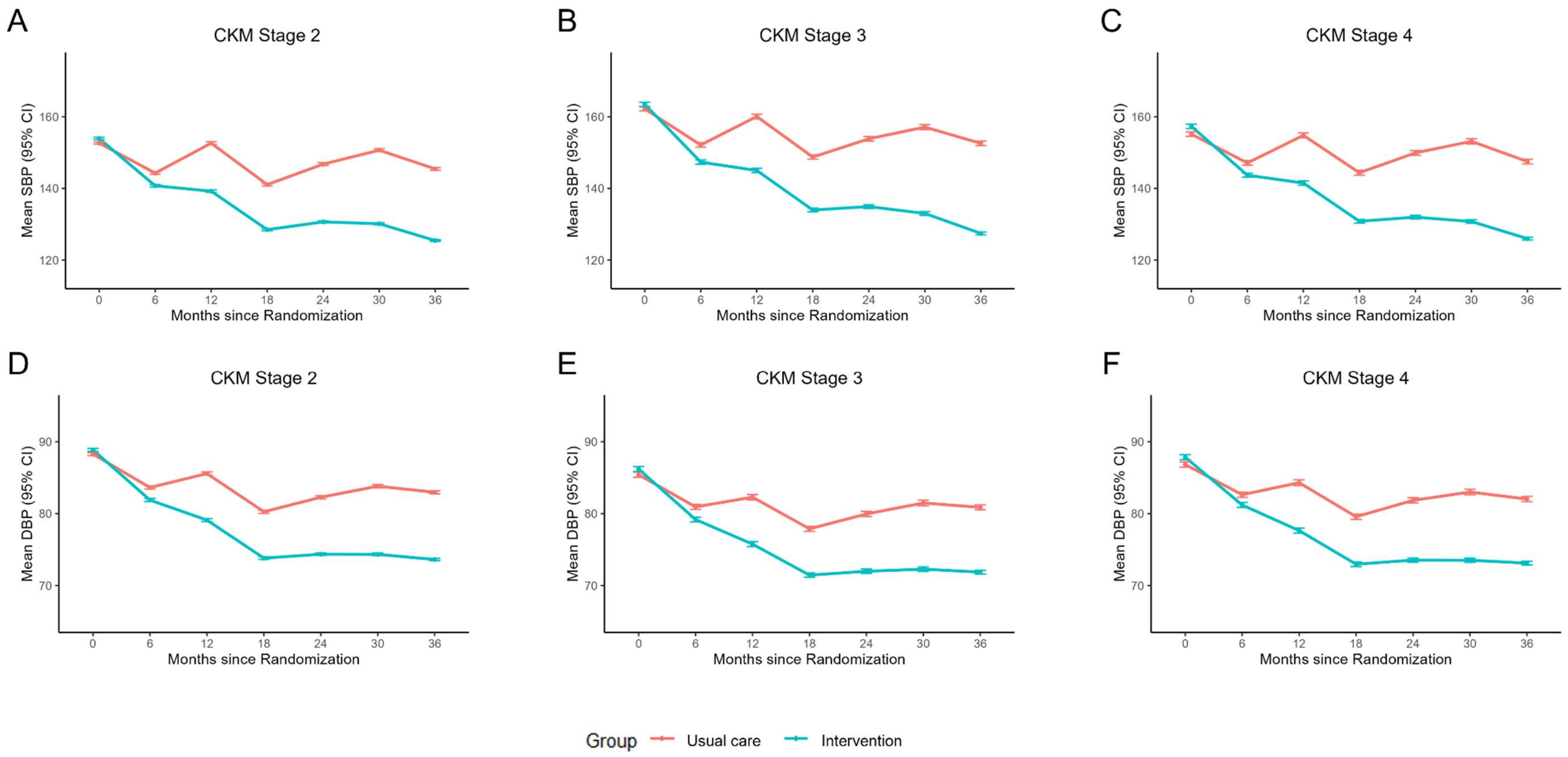

Panels A-C show mean SBP, and panels D-F show mean DBP over time for CKM stages 2 (A, D), 3 (B, E), and 4 (C, F).

Abbreviation: CKM= cardiovascular-renal-metabolic, SBP= Systolic Blood Pressure, DBP= Diastolic Blood Pressure, CI= Confidence Interval.

© 2026 Guo X et al. *JAMA Network Open*.

eFigure 3. Kaplan-Meier Curves for Myocardial Infarction in Patients With Different CKM Syndrome Stages

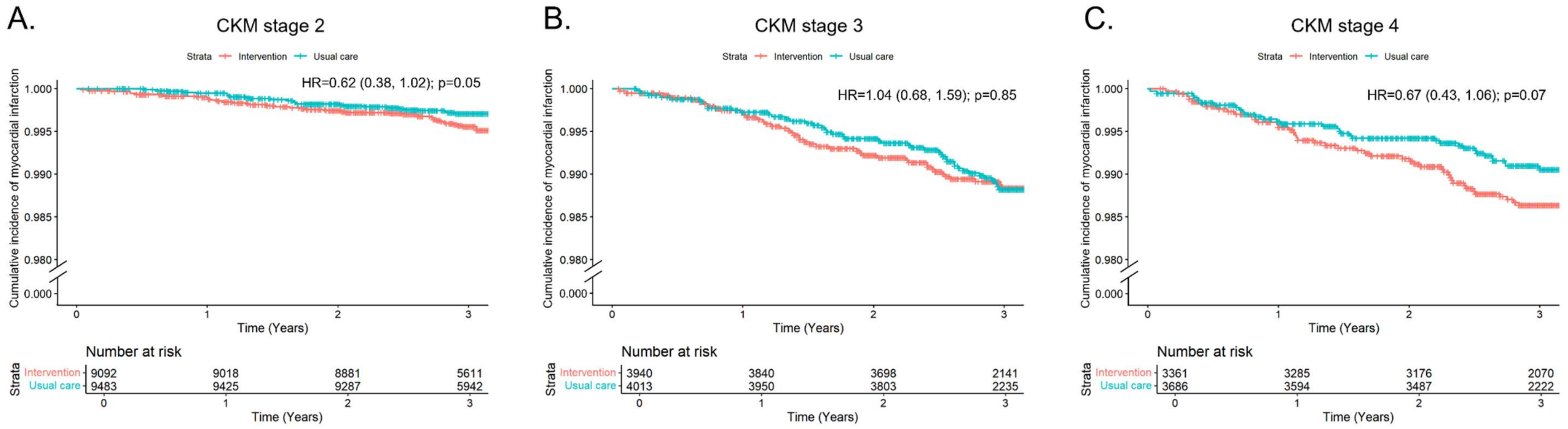

Panels: (A) CKM Stage 2, (B) Stage 3, (C) Stage 4.

Abbreviation: CKM= cardiovascular-renal-metabolic, HR= Hazard Ratio.

© 2026 Guo X et al. *JAMA Network Open*.

eFigure 4. Kaplan-Meier Curves for Stroke in Patients With Different CKM Syndrome Stages

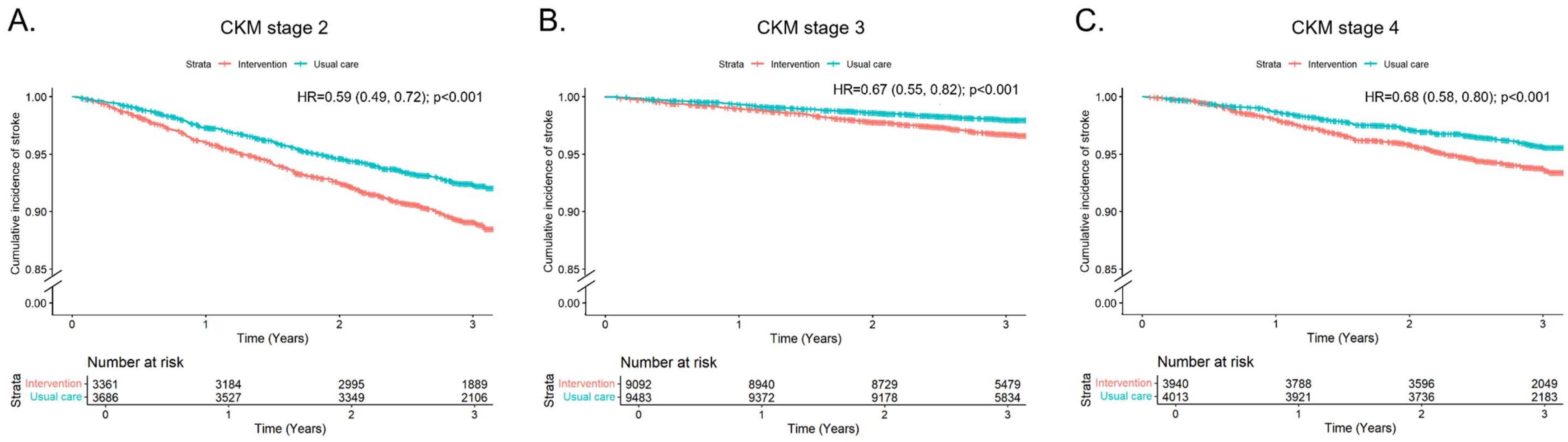

Panels: (A) CKM Stage 2, (B) Stage 3, (C) Stage 4.

Abbreviation: CKM= cardiovascular-renal-metabolic, HR= Hazard Ratio.

© 2026 Guo X et al. *JAMA Network Open*.

eFigure 5. Kaplan-Meier Curves for Heart Failure in Patients With Different CKM Syndrome Stages

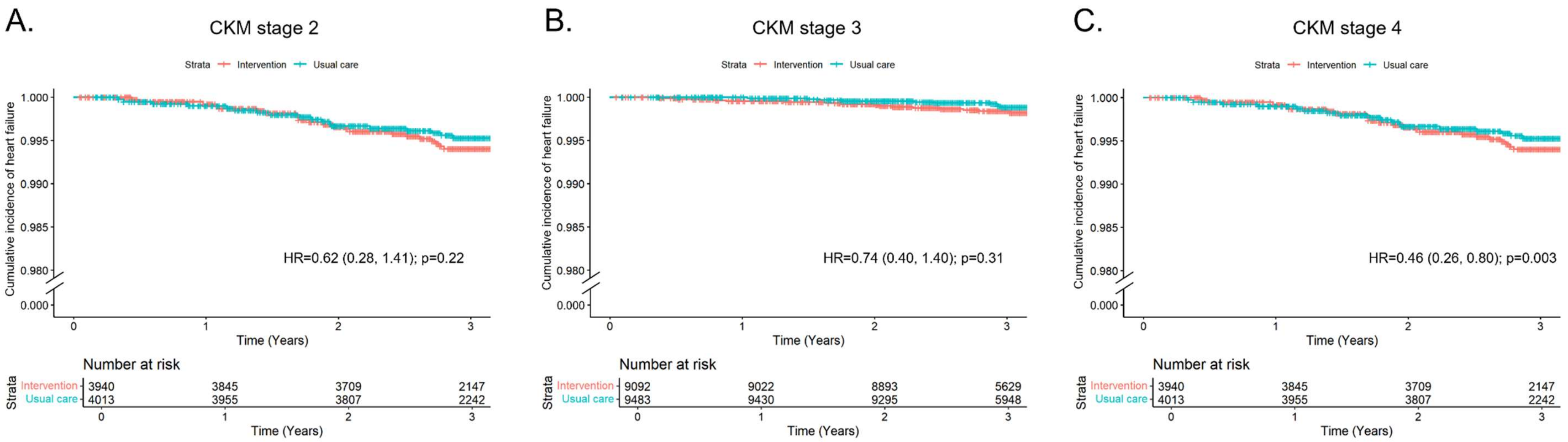

Panels: (A) CKM Stage 2, (B) Stage 3, (C) Stage 4.

Abbreviation: CKM= cardiovascular-renal-metabolic, HR= Hazard Ratio.

© 2026 Guo X et al. *JAMA Network Open*.

eFigure 6. Kaplan-Meier Curves for Cardiovascular Death in Patients With Different CKM Syndrome Stages

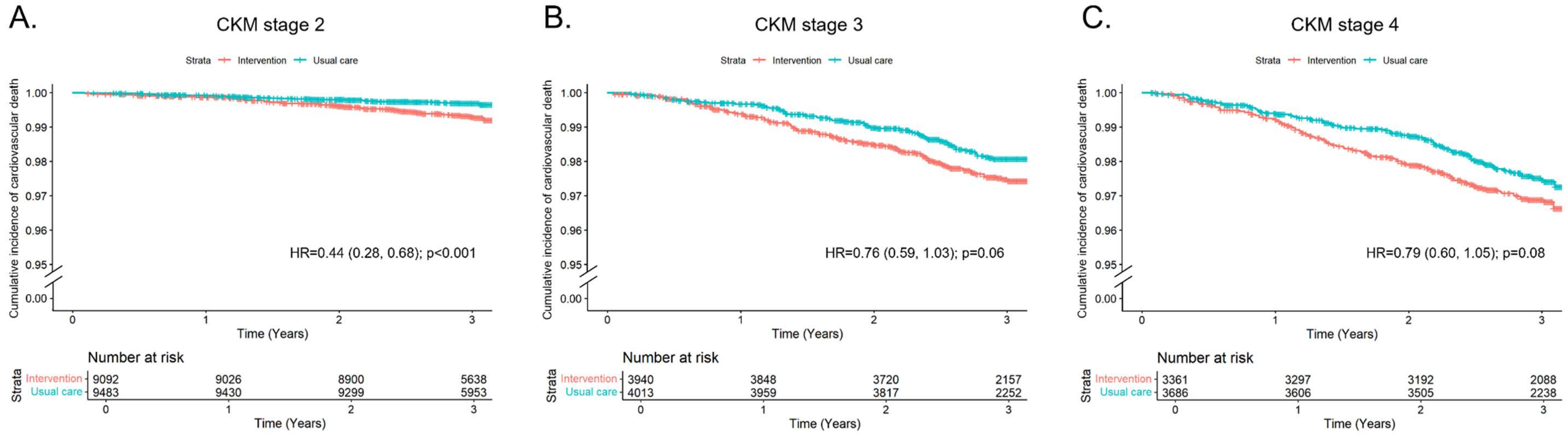

Panels: (A) CKM Stage 2, (B) Stage 3, (C) Stage 4.

Abbreviation: CKM= cardiovascular-renal-metabolic, HR= Hazard Ratio.

© 2026 Guo X et al. *JAMA Network Open*.

eFigure 7. Forest Plot of Major Cardiovascular Outcomes According to Subgroups in Participants With Different CKM Syndrome Stages

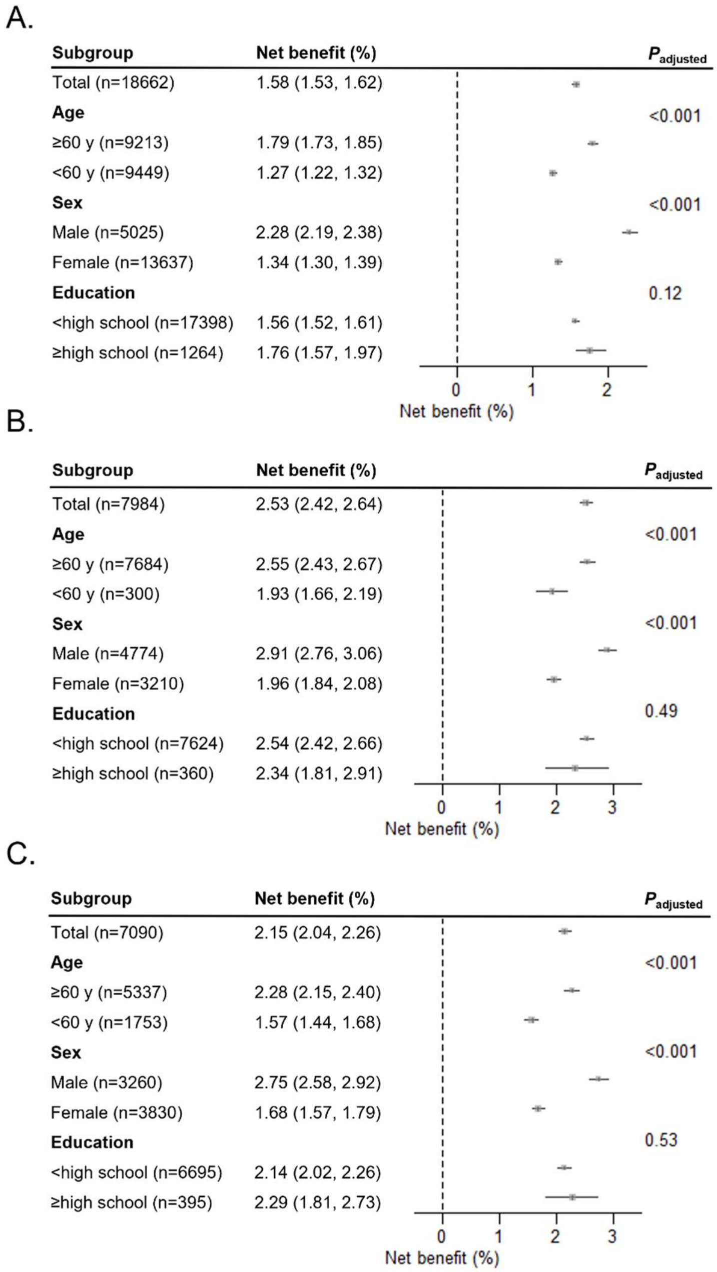

Panels: (A) CKM Stage 2, (B) Stage 3, (C) Stage 4.

Abbreviation: CKM= cardiovascular-renal-metabolic, CI= Confidence Interval.

Subgroup analyses for major cardiovascular outcomes were performed using marginal Cox models to examine potential effect modification by baseline characteristics, and multiplicative interactions were tested. Models were adjusted for province, county, and township as fixed effects and village as a random effect. P values were corrected using the Benjamini-Hochberg false discovery rate (FDR) method to account for multiple comparisons.

© 2026 Guo X et al. *JAMA Network Open*.

eFigure 8. Forest Plot of All-Cause Death According to Subgroups in Participants With Different CKM Syndrome Stages

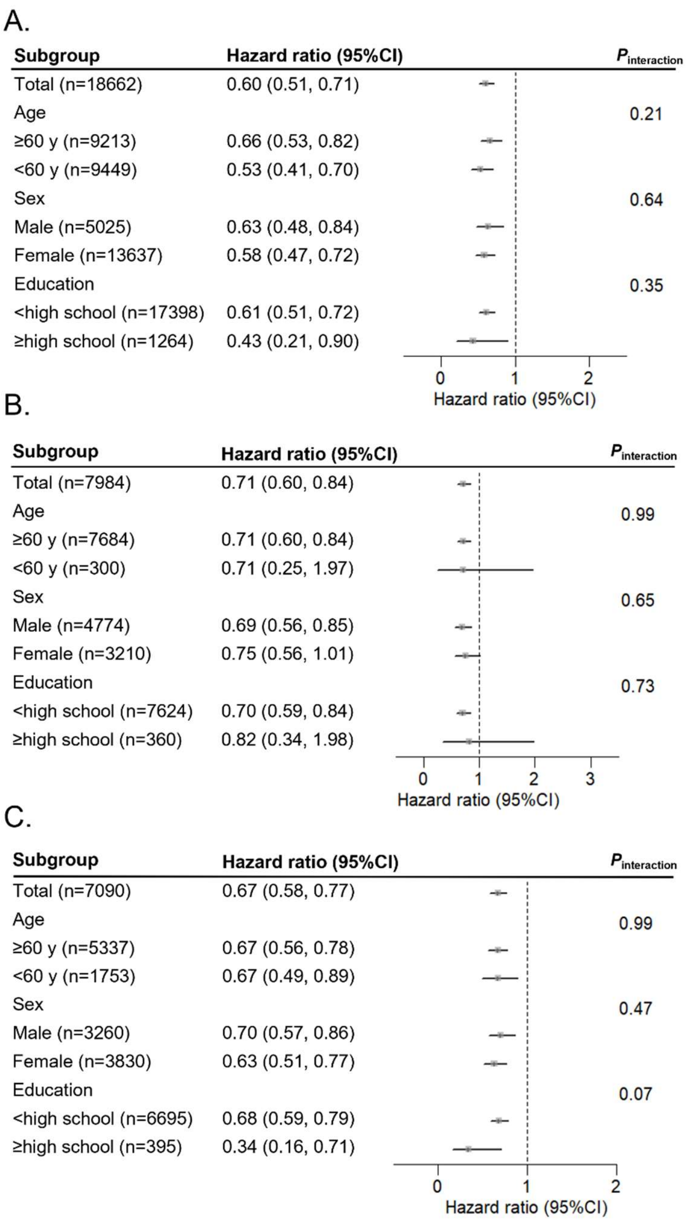

Panels: (A) CKM Stage 2, (B) Stage 3, (C) Stage 4.

Abbreviation: CKM= cardiovascular-renal-metabolic, CI= Confidence Interval.
Subgroup analyses for all-cause death were performed using marginal Cox models to examine potential effect modification by baseline characteristics, and multiplicative interactions were tested. Models were adjusted for province, county, and township as fixed effects and village as a random effect. P values were corrected using the Benjamini-Hochberg false discovery rate (FDR) method to account for multiple comparisons.

© 2026 Guo X et al. *JAMA Network Open*.

eFigure 9. Forest Plot of Net Benefits According to Subgroups in Participants With Different CKM Syndrome Stages

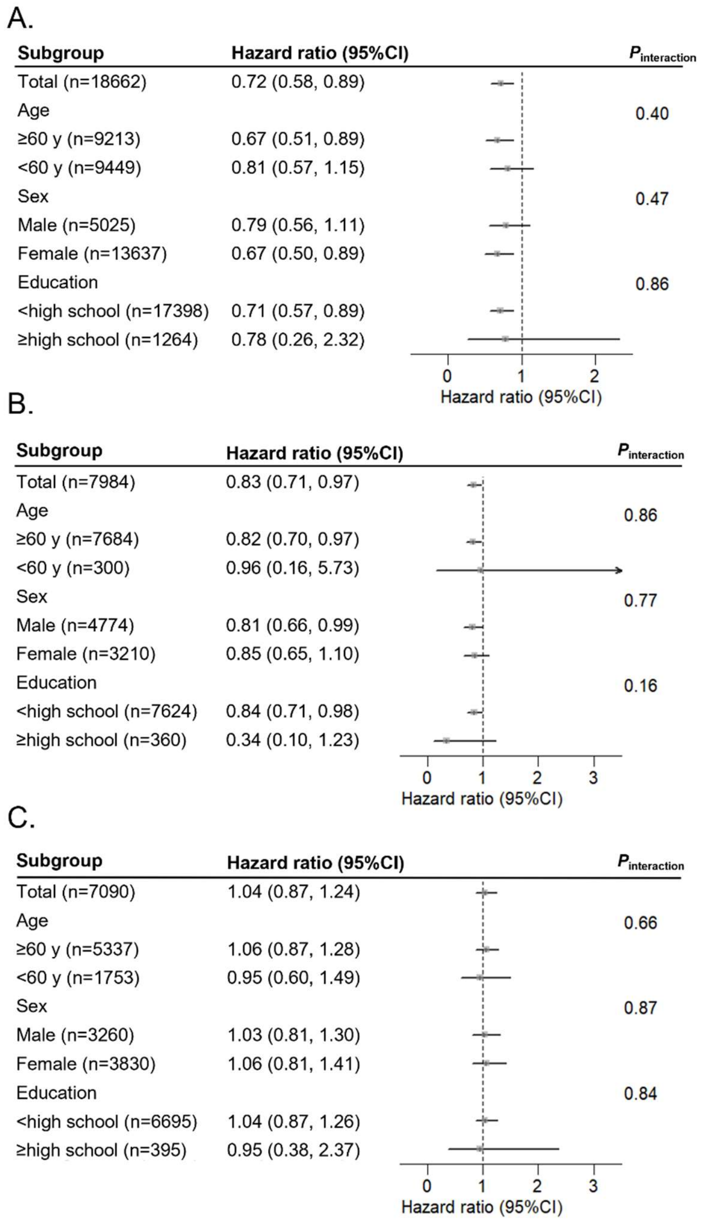

A. Net benefits in patients with CKM syndrome stage 2; B. Net benefits in patients with CKM syndrome stage 3; C. Net benefits in patients with CKM syndrome stage 4.
Abbreviation: CKM= cardiovascular-renal-metabolic.
To determine whether net benefits differed significantly between subgroups, 1,000 random variates of the net benefit were simulated within each subgroup, and Student's t-tests were used to compare between-subgroup mean values. P values were corrected using the Benjamini-Hochberg false discovery rate (FDR) method to account for multiple comparisons.

© 2026 Guo X et al. *JAMA Network Open*.

eFigure 10. Net Benefit Analysis Comparing Intensive and Standard Blood Pressure Control With Different Weights for Harm Outcomes

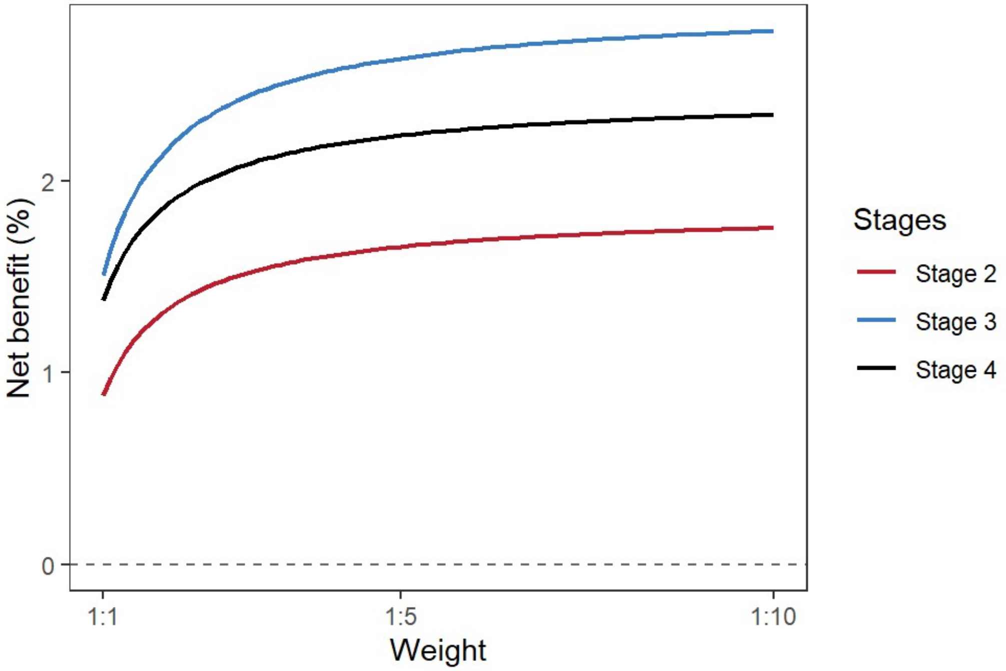

The harm-to-benefit weight ranges from 1:1 to 1:10.

© 2026 Guo X et al. *JAMA Network Open*.
