# Supplementary material for: Intensive Blood Pressure Control and Cardiovascular Outcomes Across Cardiovascular-Kidney-Metabolic Syndrome Stages: A Post Hoc Analysis of the China Rural Hypertension Control Project
Source: JAMA Netw Open. 2026 Feb 13;9(2):e2557180. doi: 10.1001/jamanetworkopen.2025.57180 (PMC12905657; doi:10.1001/jamanetworkopen.2025.57180)
Supplement: Supplement 3. — Data Sharing Statement [file jamanetwopen-e2557180-s003.pdf]

Data Sharing Statement

Guo. Net Benefit of Intensive Blood Pressure Control Across Cardiovascular-Kidney-Metabolic Syndrome Stages. *JAMA Netw Open*. Published February 12, 2026.
doi:10.1001/jamanetworkopen.2025.57180

Data
Additional Information: The China Rural Hypertension Control Project (CRHCP) is registered with ClinicalTrials.gov, NCT03527719.
Data available: Yes
Data types: Deidentified participant data, Participant data with identifiers, Data dictionary
How to access data: Data from this study can be requested from Prof Yingxian Sun (yxsun@cmu.edu.cn) after the publication of this study. Deidentifed participant data, the data dictionary, and other specified data sets can be requested, The study protocol, statistical analysis plan, and informed consent form will also be made available upon request. Specific requests for data will require the submission of a proposal with a valuable research question as assessed by the study steering committee and might require a data access agreement to be signed.
When available: With publication

Supporting Documents
Document types: None

Additional Information
Who can access the data: Researchers whose proposed use of the data has been approved
Types of analyses: Specific requests for data will require the submission of a proposal with a valuable research question as assessed by the study steering committee and might require a data access agreement to be signed.
Mechanisms of data availability: Specific requests for data will require the submission of a proposal with a valuable research question as assessed by the study steering committee and might require a data access agreement to be signed.
